# Endometriosis Is Undervalued: A Call to Action

**DOI:** 10.3389/fgwh.2022.902371

**Published:** 2022-05-10

**Authors:** Katherine Ellis, Deborah Munro, Jennifer Clarke

**Affiliations:** ^1^Department of Mechanical Engineering, University of Canterbury, Christchurch, New Zealand; ^2^Faculty of Health, University of Canterbury, Christchurch, New Zealand

**Keywords:** endometriosis, funding, women's health, quality of life, chronic pain

## Abstract

Endometriosis is an inflammatory chronic pain condition caused by uterine tissue growing outside of the uterus that afflicts at least 11% of women (and people assigned female at birth) worldwide. This condition results in a substantial burden to these women, and society at large. Although endometriosis was first identified over 160 years ago, substantial knowledge gaps remain, including confirmation of the disease's etiology. Research funding for endometriosis is limited, with funding from bodies like the National Institutes of Health (NIH) constituting only 0.038% of the 2022 health budget—for a condition that affects 6.5 million women in the US alone and over 190 million worldwide. A major issue is that diagnosis of endometriosis is frequently delayed because surgery is required to histologically confirm the diagnosis. This delay increases symptom intensity, the risk of central and peripheral sensitization and the costs of the disease for the patient and their nation. Current conservative treatments of presumed endometriosis are pain management and birth control. Both of these methods are flawed and can be entirely ineffective for the reduction of patient suffering or improving ability to work, and neither addresses the severe infertility issues or higher risk of certain cancers. Endometriosis research deserves the funding and attention that befits a disease with its substantial prevalence, effects, and economic costs. This funding could improve patient outcomes by introducing less invasive and more timely methods for diagnosis and treatment, including options such as novel biomarkers, nanomedicine, and microbiome alterations.

## Introduction

Endometriosis is a chronic inflammatory disease (1) that causes significant morbidity (2), and affects 10–15% of women of reproductive age globally (3–5). Conservatively, 1 in 9 women of reproductive age has endometriosis in the United States (US) (6) and Australia (7). Endometriosis causes tissue from the uterus to migrate and implant in other regions of the body (8, 9). This tissue interacts with the body's endocrine, musculoskeletal, vascular, reproductive, and nervous systems (10) causing numerous painful symptoms and physiological changes. There are three key types of endometriosis: superficial peritoneal, ovarian, and deep infiltrating (11). While peritoneal is the predominant presentation of the disease, ovarian affects 17–44% of endometriosis patients (12) and is characterized by the development of ovarian endometriomas, cystic lesions filled with dark endometrial fluid (13). Deep infiltrating endometriosis affects ~20% of endometriosis patients (14) and is considered the most severe form (15). Each endometriosis subtype is thought to have a different pathogenesis (16), but no etiology is confirmed (17) that explains all disease manifestations (18).

## Symptom Burden

### Symptoms

Misplaced endometriotic tissue causes a wide range of symptoms, including chronic pelvic pain, dysmenorrhea (menstrual pain), dyspareunia (painful sex), dysuria (painful urination), dyschezia (painful defecation) (19), metrorrhagia (mid-cycle bleeding), diarrhea, constipation, infertility (20), and myofascial pain, among others (1). Furthermore, the gastrointestinal symptoms of endometriosis patients are more severe than those of controls (21), which often results in both coexistence and misdiagnosis of irritable bowel syndrome (22). As the disease progresses, patients risk developing adhesions, fibrous scar tissue bands that can abnormally bind pelvic and abdominal organs (9). Endometriosis is the most frequent cause of adhesions in women and common areas for endometriosis adhesions include the anterior abdominal wall, bladder, and uterus (23). Adhesions can cause anatomical distortion, which can hinder fertility, cause rectal constriction, and be a cause of dyspareunia. In a 2019 study, the presence of endometriosis-associated adhesions was shown to significantly negatively impact quality of life (23).

The cumulative effect of these chronic pain symptoms is a substantial burden on sufferers (20) and 70% of patients live with unresolved pain (2), with impacts to all aspects of their quality of life (24). Research shows that endometriosis patients also have significantly higher rates of co-morbidities than control populations (25). The symptoms of endometriosis, particularly those associated with pain, increase the rates of chronic stress, anxiety, depression and decreased quality of life among endometriosis patients compared with those without the disease (26).

There is a well-established delay from symptom onset to diagnosis of 4–11 years for endometriosis patients (1). There are many reasons for this delay, including the lack of a unique symptom profile (27), the variety of symptoms (28) and large waitlists for the laparoscopies used to diagnose endometriosis (2). Many patients find it necessary to “doctor shop” to find a medical practitioner who will support their efforts to obtain an endometriosis diagnosis. In a 2004 study, 47% of endometriosis patients had seen at least five doctors before getting an endometriosis diagnosis or referral (29). This may be partially explained by the results of a 2021 French study, where 25% of general practitioners did not think they knew enough about endometriosis for their clinical practice (30). In a 2012 study of 173 endometriosis patients in Austria and Germany, 74.3% had experienced a misdiagnosis. These misdiagnoses included intolerances, appendicitis, irritable bowel syndrome, and pelvic inflammatory disease (31).

### Pain

In addition to painful symptoms, patients can be subject to central and peripheral sensitization (10). Central sensitization is the abnormal processing of sensory signals (32) that results in exaggerated experiences of painful and non-painful stimuli (10) through enhanced pelvic nociception. Peripheral sensitization lowers the body's threshold for nociceptor activation with repetitive and prolonged stimulation, as occurs in endometriosis (10). The combined effect of these phenomena is that over time non-painful stimuli can produce incredibly painful signals in sensitized patients.

Women with chronic pelvic pain, with or without a confirmed diagnosis, show significantly lower pain tolerances than controls (33). The severity of the decrease in pain tolerance corresponds to the duration of symptoms (33) supporting the theory that delayed diagnosis increases patient sensitization. An Australian study found endometriosis patients have significantly higher functional pain disability (pain interference with daily activities like sleep, relationships and work) than women without endometriosis (34). Furthermore, women have higher pain sensitivity than men (35, 36) as a result of complex interactions in women of anatomical, hormonal, physiological, and psychological factors (37).

### Cancer Associations

Endometriosis is a non-neoplastic invasive disease (38), although there is evidence to suggest a positive association between endometriosis and ovarian cancer (39). There is molecular evidence to suggest endometriotic lesions can undergo a transformation to clear cell and endometrioid ovarian cancers (40). This connection is controversial, and like many aspects of endometriosis, requires much more study to fully outline the potential mechanisms involved. The indication is that endometriosis increases ovarian cancer risk (19) from 1.3% in the general female population to 1.8% of endometriosis patients (41).

### Infertility

In addition to the extensive pain symptoms endometriosis patients experience, endometriosis patients have a high prevalence of infertility and sub-fertility among their cohort. Half of endometriosis patients suffer from fertility issues (42), and up to half of women with unexplained infertility or sub-fertility are subsequently found to have endometriosis (43, 44). The high rates of endometriosis interfering with fertility may relate to factors including anatomical distortions (45), diminished ovarian reserve, chronic inflammation and compromised endometrial receptivity (42).

## Lack of Funding

Endometriosis is a condition that impacts not only patients, but their families, jobs, societies, and countries. The authors believe the present issues with diagnosing, treating and funding endometriosis result from many years of misunderstanding and ignoring important female health topics. Improving funding for endometriosis research could improve the understanding of the condition, eliminate knowledge gaps, reduce time to diagnosis, expand available treatment options, improve pain management and place a long-overdue emphasis on predominantly female experiences of illness.

The National Institutes of Health (NIH) is the largest source of biomedical research funding globally, allocating $41.7 billion USD annually (46). In 2022, the expected funding allocation for endometriosis is $16 million (47), 0.038% of the budget. Since the conservative estimate is that endometriosis affects 11% of US women in their lifetime, only $2.00 per patient per year is allocated. As a comparison, 12% of US women are expected to suffer from diabetes in their lifetime (48). If it is assumed that half of the allocated diabetes research budget was for female sufferers, there is a funding allocation of $31.30 per woman, over 1,500% more than for endometriosis.

Crohn's disease, like endometriosis, is a chronic inflammatory condition (49). Crohn's disease affects the digestive tract lining, resulting in abdominal pain, weight loss, diarrhea, and fatigue (50). There are over 690,000 people with Crohn's disease in the US, or 0.21% of the population (51). In 2022, Crohn's disease research will receive $90 million in funding, $130.07 per patient, over 65 times more per patient than for endometriosis. This comparison is not to suggest Crohn's disease is overfunded, but that endometriosis is seriously underfunded.

## Economic Burden of Endometriosis

The burden of endometriosis on individual patients is substantial (20) both before and after diagnosis (52). The impact of ongoing pain can cause some patients to lose their jobs or their partners (53). Additionally, the financial burden is significant. Endometriosis patients have significantly higher healthcare resource utilization, and direct and indirect healthcare costs than controls. Endometriosis patients in the US spend $26,305 USD more than controls on healthcare expenses in the 5 years before and after diagnosis (52). In the year after diagnosis patients with endometriosis spend on average 3.5 times the amount on healthcare than controls do (25). The direct costs of endometriosis include in and outpatient treatment, surgery, and prescription costs, which in the US average $12,118 per patient, per year (54). Indirect costs, including days of work lost and reduced quality of work, were almost $16,000 per patient per year (54). In a study across ten countries lost productivity costs were generally double those of healthcare costs (55) as the average patient loses 6.4 h of work a week to presenteeism (reduced effectiveness while working) (56). Endometriosis patients begin to suffer from their condition at a young age, during a very productive period of their lives. The additive effects of fatigue, productivity loss, and time removed from the workforce, schooling and training create an immense barrier to patients being able to effectively progress in life, take up career opportunities, and in their capacity to save their earnings.

The total US endometriosis economic burden is estimated to be as high as $78–119 billion annually (54, 57). In Australia, the annual cost of endometriosis was estimated to be $16,970–20,898 per woman, per year, with 75–84% of the total due to productivity losses (58). Delays until endometriosis diagnosis increase not only the number of pre-diagnosis endometriosis symptoms but also emergency visits, hospitalizations, and overall healthcare costs (59). Compared to short delays of less than a year, long delays of 3–5 years from first symptom presentation to diagnosis, increased the cost of healthcare in the 5 years prior to diagnosis by $12,971–34,460 (59).

Lost workdays are also higher among endometriosis patients than control populations (25). In Australia, where the annual economic burden of endometriosis is estimated to be $6.5 (58) to $7.4 billion (60), endometriosis patients used on average 60% of their sick leave to treat their chronic pain (60). In a 2022 study, 65% of an Australian cohort of endometriosis patients used unpaid leave to manage their endometriosis symptoms, 64% felt judged in the workplace for their symptoms, and one in seven reported being fired as a result of their condition (61).

Furthermore, research shows there are immense productivity losses due to endometriosis for women in the workforce, even while at work. Fatigue is more common among endometriosis patients, than in control populations (62). In a 2021 Canadian study on fatigue, endometriosis patients reported substantial impairments to their work productivity with 46.5% overall work impairment due to endometriosis-related symptoms (63). These findings were like a 2013 Danish study that found that patients with endometriosis had significantly more pain than controls, were in more pain when using their sick days and used more sick days (64). This study also found that many women were embarrassed by their symptoms, felt obligated to use their sick days and often felt unable or too tired to do a satisfying job (64).

In the US, the diabetes economic burden is $327 billion (65), and with 37.3 million Americans with diabetes (48), that accounts for $8,767 of burden per patient. By comparison, the estimated economic burden of endometriosis in the US would account for $9,754–14,881 per patient, 11–70% higher than for diabetes. Thus, it is evident to the authors there is an immense financial burden not only on endometriosis patients but on nations with patients who then require high levels of healthcare utilization. These patients frequently cannot participate in their workplaces and economies to the degree they wish because of symptoms, incurring a further cost to patients and society. If endometriosis was funded by the NIH at the same level as diabetes with respect to the annual economic burden, endometriosis funding would need to increase to $298.8–455.3 million, rather than the current $16 million.

## The Present Options

Low research funding for endometriosis research means knowledge gaps are not being filled, making the development of effective diagnosis and treatment options more complicated, more time consuming, and less enticing for researchers. As a consequence, presently available options to treat endometriosis are severely limited. There are also high recurrence rates of symptoms and disease for current interventions (66). Recurrence of symptoms for non-surgical therapies, such as birth control and pain management, are rapid (18), because non-surgical treatments reduce or repress symptoms, but do not cure the disease. Furthermore, these methods are entirely inefficacious for endometriosis-associated fertility issues (19). Effective, non-invasive, non-hormonal treatments are required but are not currently available to the over 190 million global endometriosis patients (67).

### Birth Control

Birth control is a standard endometriosis treatment (68). Endometriosis birth control methods include intrauterine progesterone devices, progestin injections and combined hormone pills (69). Combined treatments increase the risk of thromboembolism, nausea and breast tenderness. Progestin injections can cause weight gain, decreased bone density, worsened acne, and depression (69). Birth control is also a limited treatment for endometriosis, as many women cannot use birth control because the side effects are too severe or because of a desire to get pregnant.

### Pain Management

Pain is the most common symptom of endometriosis (70). However, endometriosis pain management is complex. There is inconclusive evidence that non-steroidal anti-inflammatory drugs provide greater relief than placebos (71). Opioids are not a recommended treatment for endometriosis (72); however, in a cohort of 113,506 endometriosis patients in the US, 89% were utilizing opioids to manage their pain (25). Chronic opioid use can significantly increase healthcare costs for endometriosis patients compared to non-chronic users (73). Long-term opioid use for non-cancerous chronic pain, such as endometriosis, is controversial and results in an absolute adverse event rate of 78% (74). The high use of opioids among this cohort is indicative of the intensity of the pain experienced, but this approach can lead to addiction and side effects, including constipation, nausea, confusion and drowsiness (75). The required dosage to manage pain also increases with chronic use as the body becomes habituated to it (76).

### Surgery

Laparoscopic surgery is considered the “gold-standard” for diagnosing and treating endometriosis (18) and is the only method available to “confirm” endometriosis histologically (77) which provides a clear and unambiguous diagnosis for patients that is often essential for practitioners to provide the best treatment plan. According to one study, 42% of patients have undergone at least three surgeries (2). Surgery is thus an impermanent solution for many patients, with recurrence of both symptoms and lesions (19) expected for 40–50% of patients within 5 years (78), and this repeated intervention can exacerbate pain and fertility issues (79). Furthermore, surgery is a trauma to the body that activates adrenergic signaling, suppresses cell-mediated immunity and promotes angiogenesis (80). In mice with induced endometriosis, subsequent surgery increased lesion weight and microvessel density (80), which is counteractive to the intent of surgery for endometriosis.

## Evolving Possibilities

Earliest descriptions of endometriosis date back to 1860 (81) and 1920 (82). However, we still do not understand its etiology (70), the biology and function of both healthy female and endometriotic peritoneum, or the actions of endometrial stem cells (83). A substantial amount of knowledge still needs to be collected, collated, and applied to patient care. The lack of progress despite the relatively high volume of papers published about endometriosis indicates the complexity of endometriosis and the limited global funding available (83). Despite these issues, endometriosis research has been undertaken by talented researchers, and there are many promising avenues for further endometriosis research.

### New Biomarker Analysis

One of the key aspects impacting the diagnosis and treatment of endometriosis is the lack of non-invasive diagnostic tools. Biomarkers present an appealing option for non-invasive diagnosis of endometriosis. However, many biomarkers that have been assessed previously could only discern advanced disease, indicating a need for more research to locate biomarkers that can diagnose “milder” cases of the disease (84). In a 2021 study, the researchers found patients with endometriosis had distinct microbial communities in their peritoneal fluid and feces compared to the control group. In the peritoneal fluid of endometriosis patients, there were more pathogens, while there was a loss of protective microbes in feces samples (85). The authors concluded that *Ruminococcus* in the gut and *Pseudomonas* in the peritoneal fluid may be able to act as auxiliary diagnostic tools for endometriosis with further investigation into the interactions of micro-organisms and endometriosis required (85).

Follicular fluid can be obtained from follicles by fine-needle aspiration following oocyte removal (5). Researchers have found endometriosis patients have dysregulated cytokine profiles in their follicular fluid with significant upregulation of IL-1β and IL-6 (86). Conversely, the concentration of IL-12, an anti-inflammatory cytokine, inflammatory cytokine IL-10 and E-cadherin levels were lower among endometriosis patients compared to controls (5). In a 2021 study, the measurement of IL-10 in follicular fluid was able to perfectly differentiate between endometriosis patients and controls (5).

### Nanomedicines

One technology in its infancy for the treatment of endometriosis is the use of nanoparticles to aid in the imaging of, directly treating or delivering drugs to treat endometriosis (87). The key limitation for this emerging technology is that the etiology and pathogenesis of endometriosis are unknown (87). Despite this, investment in nanomedicines for endometriosis could substantially augment the capacity to diagnose and treat endometriosis. Nanoparticles have shown a capacity to accumulate in endometriotic lesions (87), which could improve the use of imaging technologies to diagnose endometriosis. This technology could also provide a method for targeting endometriotic lesions without the requirement of surgery. Potential drugs that could be delivered by nanotechnological methods could be anti-inflammatory, antioxidant, anti-angiogenic and immunomodulating molecules (88), which may have the capacity to reduce the size of or eliminate endometriosis lesions, rather than just suppress symptoms. However, much more pre-clinical and clinical research is required to support the use of this emerging technology for endometriosis (88).

### Alterations to the Microbiome

Imbalances to gut microbiota composition have been connected to the compromised immunosurveillance and altered immune profiles associated with endometriosis (89), with animal studies consistently showing the impact of the gut microbiota on endometriosis and endometriosis on gut microbiota (90). In addition to being a potential site for novel biomarkers, the gut microbiota may be a target site for new treatments. In a 2019 study by Chadchan et al., mice with induced endometriosis were subjected to antibiotic therapies (91). Broad-spectrum antibiotics were shown to significantly reduce lesion size and inflammatory response. Furthermore, the authors showed that fecal transfer from mice with endometriosis restored lesion growth and inflammation in mice treated with the antibiotic metronidazole (91). Conversely, metronidazole-treated mice that received fecal transfers from mice without endometriosis had significantly smaller lesions, suggesting a role for the gut microbiome in the progression of endometriosis (91). The effect of gut microbiota on endometriosis is not solely negative. The bacteria-derived metabolite n-butyrate is a short-chain fatty acid that is significantly downregulated in mice with induced endometriosis. In a 2021 study, n-butyrate treatment significantly reduced lesion growth and inflammatory cell infiltration in a mouse model (92). Therapies that address endometriotic alterations to the gut microbiota could have immense potential to reduce the growth of lesions and the effects of inflammation for endometriosis patients.

## Discussion

Despite progress, critical gaps remain in the fundamental understanding of endometriosis. This means there are opportunities to substantially expand and improve our core understanding of this important health topic. The authors feel endometriosis warrants more attention to fill these fundamental knowledge gaps. There are not enough people working in this vital space, likely due to insufficient funding. If endometriosis was funded by the NIH at half the level of diabetes, the budget would increase almost 16 times to over $250.4 million annually. It is the belief of the authors that present levels of endometriosis funding do not reflect the immense pain of patients, long delays in diagnosis, the ineffectiveness of common treatment options, massive knowledge gaps, substantial economic burdens or the immense costs borne by individual patients. Unexplored in the scope of this paper, but vital, is the investment into structures to translate research findings into clinical care, understanding of the epidemiological underpinnings of patient diversity, increased awareness through public education about endometriosis so affected patients are better aware, and into healthcare practitioner training about how best to treat and support endometriosis patients.

There is a lot of promising research underway that could create substantial positive ramifications for patients. These include the chance for non-invasive biomarker auxiliary diagnosis methods, the application of nanoparticle drug delivery and treatments targeting the microbiome. An area of immense potential for developing new non-invasive diagnostic and treatment options may be the application of nanoparticles to deliver therapies directly to endometriotic lesions.

Advancement in the identification and treatment of endometriosis is challenging but entirely possible. It is the opinion of these authors that if endometriosis had more representative funding, the rate of advancement of non-invasive diagnostic and treatment methods could be significantly increased, with long-term benefits for patients and society.

## Data Availability Statement

The original contributions presented in the study are included in the article; further inquiries can be directed to the corresponding author.

## Author Contributions

KE is the first author of this paper through conception and literature collection. DM and JC have contributed equally to this work by critically revising and editing the article. All authors contributed to the article and approved the submitted version.

## Conflict of Interest

The authors declare that the research was conducted in the absence of any commercial or financial relationships that could be construed as a potential conflict of interest.

## Publisher's Note

All claims expressed in this article are solely those of the authors and do not necessarily represent those of their affiliated organizations, or those of the publisher, the editors and the reviewers. Any product that may be evaluated in this article, or claim that may be made by its manufacturer, is not guaranteed or endorsed by the publisher.
